# Genome-wide identification of *R-SNARE* gene family in upland cotton and function analysis of GhVAMP72l response to drought stress

**DOI:** 10.3389/fpls.2023.1147932

**Published:** 2023-07-03

**Authors:** Bingxuan Li, Gen Zhou, Yanbin Li, Xueting Chen, Huiting Yang, Yan Li, Minhua Zhu, Libei Li

**Affiliations:** ^1^ Department of Agronomy and Horticulture, Jiangsu Vocational College of Agriculture and Forestry, Jurong, China; ^2^ Key laboratory of Quality Improvement of Agriculture Products of Zhejiang Province, College of Advanced Agriculture Sciences, Zhejiang A&F University, Hangzhou, China; ^3^ College of Life Sciences, Xiamen University, Xiamen, China; ^4^ Shanghai Fisheries Research Institute, Shanghai Fisheries Technical Extension Station, Shanghai, China; ^5^ College of Life Sciences, Nanjing Agricultural University, Nanjing, China; ^6^ Basic Medicine Department, Heze Medical College, Heze, China; ^7^ College of Landscape and Architecture, Zhejiang A&F University, Hangzhou, China

**Keywords:** *G. hirsutum*, *R-SNARE* gene family, gene identification, gene expression, drought stress

## Abstract

Soluble N-ethylmaleimide-sensitive factor attachment protein receptors (R-SNAREs) mainly promoted the assembly of the SNARE complex to drive the final membrane fusion step of membrane transport. Previous research on R-SNAREs has mainly focused on development and growth and has rarely been involved in abiotic stress, especially in cotton. Here, we performed a comprehensive analysis of *R-SNARE* genes in upland cotton. In total, 51 *Gh-R-SNARE* genes across six phylogenetic groups were unevenly distributed on 21 chromosomes. Cis elements related to plant growth and response to abiotic stress responses were found in the promoter region of *Gh-R-SNAREs*. Nine *Gh-R-SNARE* genes were obviously upregulated under drought stress conditions by RNA-seq and qRT–PCR analysis. Among them, *GhVAMP72l* might be the key candidate gene contributing to drought stress tolerance in cotton by virus-induced gene silencing (VIGS) assay. These results provide valuable insights for the functional analysis of cotton *R-SNAREs* in response to drought stress and highlight potential beneficial genes for genetic improvement and breeding in cotton.

## 1 Introduction

Cotton as one of the most cash crops accounts for approximately 35% of the production of total fiber worldwide (Billah et al., 2021; Li et al., 2021). Global warming conditions have resulted in water shortages globally, and cotton is mostly planted in water-deficient areas, which makes drought one of the most serious problems faced by cotton producers. Drought stress causes an average decrease in cottonseed yield and biological yield of 42% and 55%, respectively (Abdelraheem et al., 2019). Therefore, it is urgent to explore cotton drought-responsive genes and to study the cotton drought tolerance mechanism.

Several key physiological responses of cotton to drought stress have been identified, including stomatal closure, changes in root development, cellular changes, photosynthesis changes, hormone responses, and scavenging of reactive oxygen species (ROS) (Ullah et al., 2017). Vesicle trafficking, a housekeeping process, is important for these physiological responses and can ensure the correct localization of proteins specialized in sensing drought stress stimuli and affecting the response (Xue et al., 2018; Luo et al., 2022). Vesicle fusion, which is mediated by the SNARE complex, is the final step of vesicle trafficking (Wang et al., 2017; Kwon et al., 2020). SNARE proteins have been classified as Q (glutamine)- and R (arginine)-sensitive factor attachment protein receptors (SNAREs), depending on the conserved residues contributing to the central layer formed in the complex, and Q- and soluble N-ethylmaleimide R-SNAREs are localized to target membranes and vesicles, respectively (which are also called T- and V-SNAREs, respectively) (Karnik et al., 2017; Yoon and Munson, 2018; Waghmare et al., 2019). Three Q-SNARE (Qa, Qb, Qc-SNARE) motifs bind to R-SNARE motifs to form a tetrameric bundle of coiled helices, thus resulting in fusion of the vesicle and target membrane (Wang et al., 2017).

Evidence gathered over the past decade has revealed that SNARE proteins function in response to drought stress. Recently, *GsSNAP33* was successively isolated from soybean, and the overexpression lines showed tolerance to drought stress by genetic transformation of *GsSNAP33* in Arabidopsis compared with wild type, indicating its possible contribution to drought stress tolerance (Nisa et al., 2017). *GmSYP24*, a Qa-SNARE gene harboring an LEA2 domain (whose function is not clear), was identified from soybean, and the expression of *GmSYP24* was greatly and rapidly induced by drought stress. In *GmSYP24*-overexpressing transgenic soybean plants, the water content, peroxidase (POD), and superoxide dismutase (SOD) activities, and abscisic acid (ABA; a plant abiotic stress hormone)-responsive gene expression all increased compared with those of the nontransgenic controls, which means that *GmSYP24* plays an important role in drought tolerance in the ABA signal pathway (Chen et al., 2019). *Vesicle-associated membrane protein-associated protein* (*TaVAP*) was identified as a drought-inducible gene in wheat, and overexpression of *TaVAP* in Arabidopsis increased tolerance to water-stress conditions (Singh et al., 2018). Under light conditions and following Ca^2+^-induced stomatal closure, the *atsyp121* (a Qa-SNARE) mutant of Arabidopsis shows delayed stomatal opening and slowed vegetative growth, which means that SPY121 participates in the drought stress response (Eisenach et al., 2012). In cotton, Qbc-SNARE, *GhSNAP33* expression was induced by polyethylene glycol 6000 (PEG6000) treatment and enhanced drought tolerance when overexpressed in Arabidopsis (Wang et al., 2018).

In addition to Q-SNAREs, R-SNAREs are involved in drought stress responses. *AtVAMP711*, a tonoplast-specific R-SNARE in Arabidopsis, participates in the localization of ROS, and deletion of *VAMP711* results in higher plasma membrane (PM) H^+^-ATPase activity and slower stomatal closure after ABA treatment (Leshem et al., 2010; Xue et al., 2018; Xue et al., 2019). Except for VAMP711, VAMP721/722 (an R-SNARE in Arabidopsis) also reacts to ABA, and the protein levels of VAMP721/722 gradually decrease after ABA treatment, which implies that VAMP711/711/712 are involved in drought stress responses (Yi et al., 2013; Zhang et al., 2015). Despite the important roles of SNAREs in the drought stress response of plants, functional information on SNAREs is scarce, especially in cotton (Uemura and Ueda, 2014; Kwon et al., 2020; Won and Kim, 2020). There is less information available on R-SNAREs than on Q-SNAREs. Thus, little is known about the functions of R-SNARE family gene responses to drought stress in cotton (Sudhof and Rothman, 2009; Tian et al., 2021).

In this study, we identified 51 *Gh-R-SNARE* genes from upland cotton using bioinformatics, including phylogenetic trees, gene structures, cis-elements, and chromosomal location analyses. Based on transcriptome analysis and VIGS assay, our analyses indicated that *GhVAMP72l* may be involved in responses to drought stress. The results provide an important foundation for further understanding the evolution and function of R-SNARE proteins and exploiting them in cotton drought-responsive genetic improvement.

## 2 Materials and methods

### 2.1 Growth conditions of plant materials and treatment of drought stress

All upland cotton materials used in this study were ‘TM-1’ (*G. hirsutum* genetic standard strain). The seeds of ‘TM-1’ germinated and grew under the following conditions: 18–28 °C temperature and a 16/8 h light/dark photoperiod. To analyze *R-SNARE* gene expression levels under drought stress, ‘TM-1’ seedlings were grown in sterilized soil for 3 weeks, cotton was transferred to 17% PEG6000 solution for cultivation, and another portion of cotton was transferred to water for cultivation as control. Cotton leaves were collected at different time intervals after drought treatment (17% PEG6000 at 1, 3, 6, 9, 12, 24, and 48 h) for RNA extraction and reverse transcription. The ‘TM-1’ materials used to check drought resistance were subjected to 17% PEG6000 at two weeks after germination, when the cotton reached the three-leaf stage. Meanwhile, samples were also collected from ‘TM-1’ seedlings grown under normal conditions as control.

### 2.2 Identification of *Gh-R-SNARE* genes

A total of 17 R-SNARE protein sequences of Arabidopsis were downloaded from TAIR (https://www.arabidopsis.org/) as query to identify the R-SNARE genes in upland cotton. The protein sequences of upland cotton were downloaded from the COTTONGENE database (https://www.cottongen.org/) (version: HAU_v1). BLAST program was executed and confirmed by HMMER software (pfam00957). The potential *R-SNARE* genes were filtered according to the following criteria: (a) E-value ≦1e^−60^, (b) identity ≥40%, (c) alignment length ≥150, and (d) alignment score ≥250. The PFAM databases (http://pfam.xfam.org/) were used to confirm the structural integrity of the R-SNARE domain. The gene IDs of the *R-SNARE* genes in upland cotton are described in **Additional File 1** (**Table S1** **)**. Biophysical characteristics of R-SNARE proteins were analyzed with the online tool ExPASy (http://www.expasy.org/). Sequence alignments of the R-SNARE domain in upland cotton were aligned using the Geneious Prime software (https://www.geneious.com/download/). Phylogenetic analyses were carried out using the neighbor-joining method with MEGAX software by whole protein sequence, referring to previous reports (Li et al., 2019).

### 2.3 Chromosomal location and gene collinearity analysis

Chromosomal location of *R-SNARE* genes was plotted based on location information in the *G. hirsutum* genome database (version: HAU_v1) by TBtools Gene Location Visualize function (Chen et al., 2020). Coding sequence fasta and gff3 files of upland cotton (https://www.cottongen.org/) (version: HAU_v1) were used to construct collinearity gene duplication and synteny relationships by MCscan (https://github.com/tanghaibao/jcvi/wiki/MCscan-(Python-version)) between At subgenome and Dt subgenome.

### 2.4 Cis-elements, gene structure, conserved motifs, and protein domain analysis

Cis-elements in the promoter sequences of each R-SNARE gene in *G. hirsutum* were analyzed using PlantCARE software (Lescot et al., 2002). The gene structures of the *Gh-R-SNAREs* were identified by genomic and corresponding coding sequences, and the intron/exon arrangement of R-SNARE genes in *G. hirsutum* was elucidated by TBTools Gene Structure View function (Zhou et al., 2022). The conserved motifs of the R-SNAREs in *G. hirsutum* were detected by the MEME program (https://meme-suite.org/meme/).

### 2.5 Transcriptome analysis of *R-SNARE* genes in upland cotton

The expression of *R-SNARE* genes in upland cotton was analyzed by transcriptomic analysis. The public data (PRJNA490626) were obtained from the NCBI database (https://www.ncbi.nlm.nih.gov/bioproject/). The detailed information is listed as follows: (1) Multiple organs of TM-1 for each sample included root, stem, leaf, bract, anther, and various reproductive organs; (2) Leave under drought stress at five developmental stages as follows: 1 h, 3 h, 6 h,12 h, and 24 h; Each sample was performed RNA-seq with three replicates that have 150 bp paired-end reads. The expression value of each gene was determined using Salmon software (Patro et al., 2017) based on ‘TM-1’ reference genome (Wang et al., 2019) with the following parameters: (-1 -2 -p 30 -o -numBootstraps 1,000). The transcriptome data corresponding to multiple organs and drought stress were normalized to the average expression levels (log_2_) based on transcripts per kilobase million (TPM) values. A clustered heatmap of the data was constructed by TBtools.

### 2.6 RT-qPCR analysis

Approximately 0.1 g frozen leaves of upland cotton were crushed into powder in liquid nitrogen. The supernatant were collected using centrifugation and transferred to a new centrifuge tube. Then, we used the RNA Easy Fast Plant Tissue Kit to extract total RNA (Lysis Buffer, Proteinase K and RNA Easy Fast Plant Tissue Kit were brought from TIANGEN BIOTECH Co., Ltd., Beijing, China) (Liu et al., 2019). Total RNA was reversed into cDNA using the PrimeScript^TM^ RT reagent Kit with gDNA Eraser (Takara Biomedical Technology (Beijing) Co., Ltd., Beijing, China). The upland cotton gene *GhUBQ7* worked as an internal reference. Quantitative analysis was performed using AceQ^®^ qPCR SYBR Green Master Mix (Vazyme Biotech Co., Ltd., Nanjing, China) and a real-time qPCR system (ABI Step One Plus™), with three biological repeats (Li et al., 2017; Wu et al., 2017).

### 2.7 VIGS technology and drought stress treatment

A 300 bp fragment of *GhVAMP72l* was amplified from the cDNA of ‘TM-1’ and inserted into a *pTRV2* vector using the *EcoR1* and *Kpn1* restriction sites. Moreover, *pTRV::GhCLA* was constructed as a visual marker to monitor the silencing efficiency and *pTRV::0* (empty vector) was used as a negative control. All the vectors were transferred into Agrobacterium strain *GV3101*, and then the cotyledons of 10-day-old ‘TM-1’ seedlings were injected with the transformants at 25 °C. Two weeks after infiltration, the *TRV::0* plants showed an albino phenotype. When they reached the three-leaf stage, some of the cotton plants were treated with water as a control, whereas the others were treated with the same amount of 17% PEG6000 until the phenotypes became distinct (all the plants grown in soil). All the primers used in this study are listed in **Additional File 1** (**Table S2** **)**.

### 2.8 Determination of physiological parameters related to drought stress

The content of proline and malondialdehyde (MDA), the activity of catalase (CAT), and POD can reflect the metabolic state and adaptation mechanism of plants under drought stress. To measure proline content, 0.2 g fresh leaf materials of *TRV::GhVAMP72l* and *TRV::0* plants grown under normal or drought stress conditions were extracted with 2 ml ice-cold extraction solution, then kept in boiling water bath for 10 min, then centrifuged at 10,000*g* for 10 min, collected supernatant and cooled to room temperature. The supernatant was immediately used to detect the proline content using the proline quantification assay kit.

To measure MDA content, 0.1 g fresh leaf materials were thoroughly ground to powder in liquid nitrogen, samples were mixed in 1 ml ice-cold extraction solution and centrifuged at 8,000*g* for 10 min. The supernatant was used to detect the MDA content using a malondialdehyde quantification kit.

To determine the activity of CAT and POD, 0.1 g leaf samples were ground to powder in liquid nitrogen. Approximately 1 ml of ice-cold 100 mM phosphate buffered solution (pH 7.0) was added, mixed for several minutes, and then centrifuged at 8,000*g* for 10 min at 4 °C. The supernatant was collected for analyzing the activities of CAT and POD using CAT and POD Assay Kit.

The proline, MDA, CAT, and POD Assay Kits were purchased from Suzhou Comin Biochemistry Co., Ltd. (Su Zhou, China).

### 2.9 Statistical analyses

One-way ANOVA algorithm was performed using R software (version: 4.2.2) by the function of ‘aov.’

## 3 Results

### 3.1 Identification and protein characteristics of R-SNAREs in cotton

Arabidopsis encodes 17 R-SNARE/R-SNARE-like domain-containing proteins, namely 15 standard R-SNARE proteins and 2 special R-SNARE proteins containing WD40 domains (Li et al., 2019). To identify *R-SNARE* genes in *G. hirsutum*, BLAST searches were performed in which 17 R-SNARE protein sequences from Arabidopsis were used as query sequences. Then, we used the online NCBI CDD tool to check whether the predicted sequences contained the R-SNARE/R-SNARE-like domain typical of R-SNARE proteins. The candidate *R-SNARE* genes were renamed based on their genetic relationship with those of *A. thaliana* (Liu et al., 2019). A total of 51 *R-SNARE* members were identified in *G. hirsutum* [**Additional File 1** (**Table S1** **)**]. Then, we further analyzed the physical and chemical parameters of Gh-R-SNARE proteins. For ordinary R-SNAREs, those of *A. thaliana* had 124–285 amino acid (aa) residues; *O. sativa* 214–248 aa; and *G. hirsutum* 142–266 aa. For specific R-SNARE proteins, the encoded protein lengths of those in *A. thaliana, O. sativa*, and *G. hirsutum* ranged from 1,050–1,124, 701–1,101, and 1,040–1,100, respectively. Similarly, the isoelectric points of the R-SNARE proteins in *A. thaliana, O. sativa*, *and* *G. hirsutum* ranged from 5.48 to 10.24, 5.16 to 9.67, and 5.41 to 10.35, respectively. Other basic information for all R-SNARE members in *A. thaliana*, *O. sativa*, and *G. hirsutum* are listed in **Additional File 1** (**Table S3** **)**. These results indicate that the *R-SNARE* gene family among plants throughout evolution is low and that the *R-SNARE* gene family is highly conserved.

### 3.2 Phylogenetic analysis of the R-SNARE gene family

**Figure 1:**
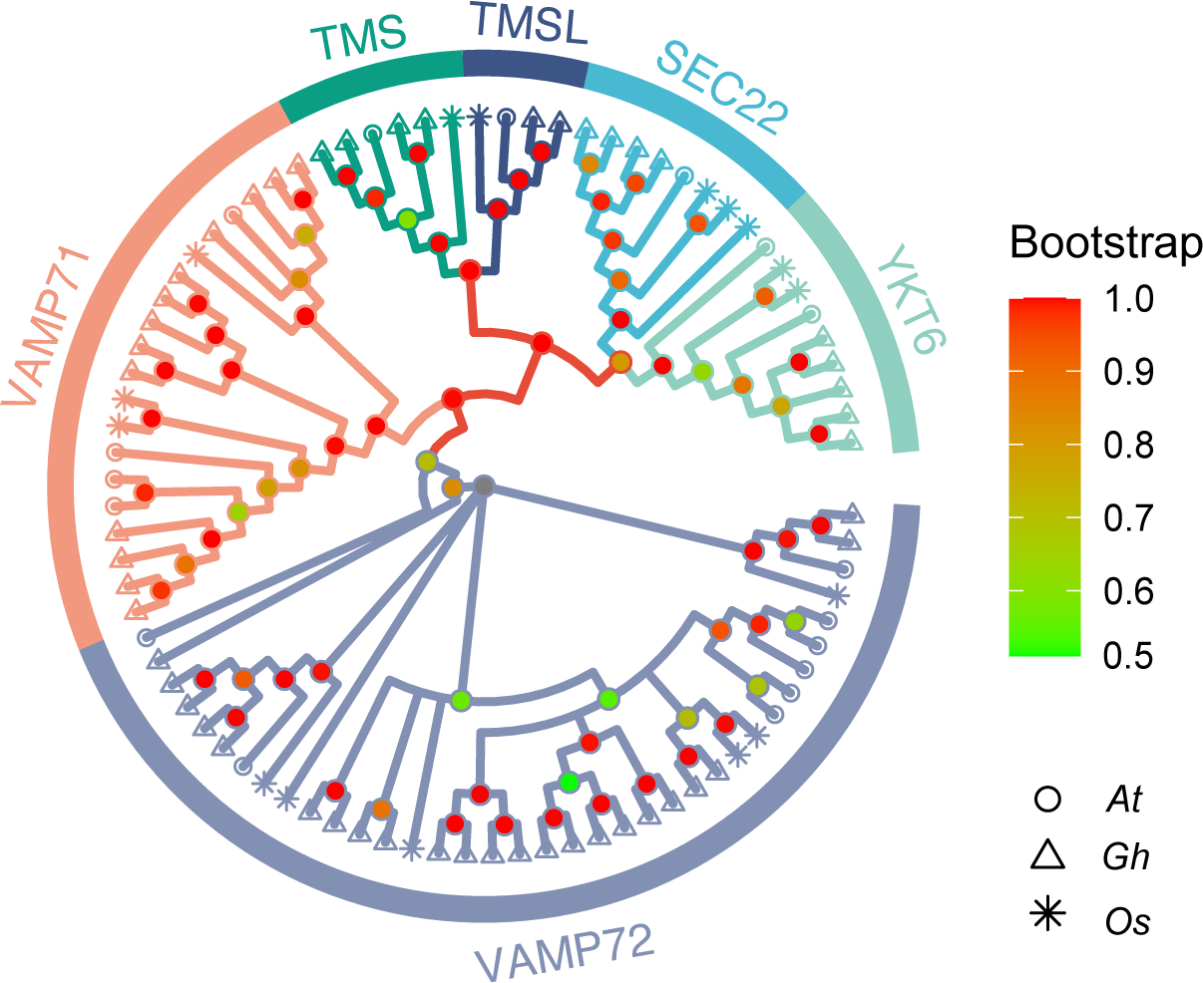
Phylogenetic relationships of R-SNARE gene families in *A. thaliana* (*At*), *O. sativa* (*Os*), and *G. hirsutum* (*Gh*). The six subgroups and three species analyzed are represented by different colors and shapes, respectively.

To study the evolutionary relationships between *R-SNARE* genes, an unrooted phylogenetic tree was generated by using the R-SNARE protein sequences from *A. thaliana*, *O. sativa*, and *G. hirsutum*. According to the phylogenetic tree, the protein sequences of *R-SNAREs* were divided into six subgroups (**Figure 1**). In reference to the results of Arabidopsis, the six subgroups were named as follows: TMSL, TMS, SEC22, YKT6, VAMP71, and VAMP72 (Lipka et al., 2007; Li et al., 2019). In addition, each subgroup has a different number of R-SNAREs: VAMP72 has 37 members, which constitutes the largest subgroup. Furthermore, the number of R-SNAREs in each subgroup from every species is roughly equal, which further indicates that the R-SNARE genes are conserved in both monocotyledons and dicotyledons. Detailed phylogenetic tree grouping information is listed in **Additional File 1** (**Table S4** **)**.

### 3.3 Collinearity and genomic distribution analysis

**Figure 2:**
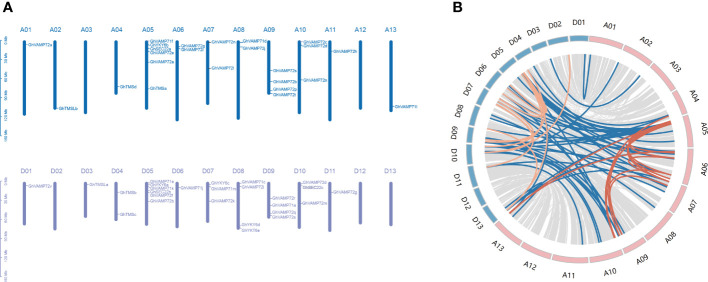
Collinearity analysis and chromosomal distribution of R-SNARE genes. **(A)** Chromosomal distribution of *R-SNARE* genes in *G. hirsutum*. The chromosomes of the At and Dt subgenomes are drawn in blue and purple, respectively. The chromosome number is located directly above each vertical bar. **(B)** Synteny blocks between the At and Dt subgenomes. The gray lines represent synteny blocks in the upland cotton genome; the red lines represent *Gh-R-SNARE* genes involved in synteny blocks between At subgenome; the orange lines represent *Gh-R-SNARE* genes involved in synteny blocks between Dt subgenome; the blue lines represent *Gh-R-SNARE* genes involved in synteny blocks between At and Dt subgenomes.

To investigate duplication events and genomic distribution of R-SNAREs in cotton, collinearity and the genomic distribution analyses were performed. In total, 50 genes were distributed throughout the 26 chromosomes, comprising 24 genes located on the At subgenome and 26 genes on the Dt subgenome (**Figure 2A**). *GhVAMP72o* was not mapped to chromosomes but to scaffolds. Chromosomes A05 and D05 had the highest number of *R-SNARE*s (six), followed by chromosomes A09, D08, and D09, each of which had four *R-SNARE* genes. The other 21 chromosomes had one to four *R-SNARE* genes, whereas chromosomes A03, A12, A13, D02, D12, and D13 contained none of the genes. These results showed that *R-SNARE* genes were widely distributed in *G. hirsutum*. In addition, we identified 47 *R-SNARE* genes in upland cotton that were involved in 70 synteny blocks, except for *GhVAMP71f*, *GhVAMP71g*, *GhYKT6c*, and *GhVAMP72o* (**Figure 2B**). Of the total synteny blocks, only 13 (18.5%) were located between the At subgenomes, and the same number were located between Dt subgenomes, probably because of segmental duplication or chromosomal rearrangement in the genomes. The majority of synteny blocks, 44 (62%), were located between the At and Dt sub-genomes, including 39 orthologous *R-SNARE* genes in upland cotton. Among these genes, *GhYKT6b* exists within eight synteny blocks, and only one pair of tandem duplications of *Gh-R-SNARE* genes (GhVAMP71i/GhVAMP71g) was located on chromosome A06.

### 3.4 Promoter cis-elements and gene structure analysis

**Figure 3:**
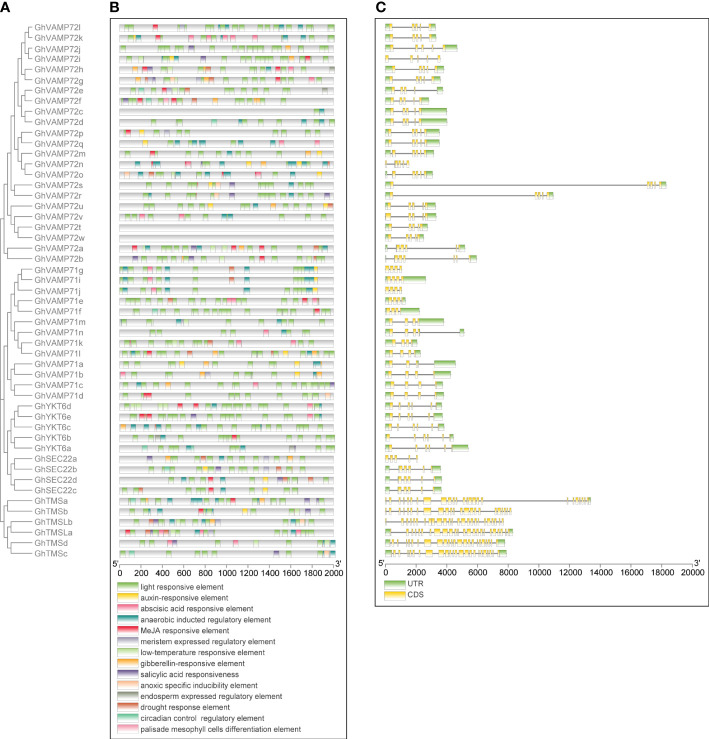
Phylogenetic analysis, predicted cis-elements, and gene structure of the R-SNARE family members in *G. hirsutum*. **(A)** Phylogenetic tree of all R-SNAREs in *G. hirsutum*. The predicted cis-elements and gene structures are presented next to their corresponding proteins. **(B)** Predicted cis-elements of R-SNARE genes. Each element is indicated by a specific color. **(C)** The exon/intron composition of *R-SNARE* genes: the yellow boxes represent exon CDSs, the black lines indicate introns, and the green boxes indicate UTRs. The gene lengths can be estimated by the scales at the bottom.

Specific sequences of a gene promoter can be combined with transcription factors to regulate gene expression related to development and stress responses (Liu et al., 2022). To further elucidate the regulatory mechanism of R-SNARE expression, the 2 kb sequence upstream of the start codon of the R-SNAREs in *G. hirsutum* was selected to analyze the constitution of cis-elements. All R-SNARE genes used for the analysis were arranged according to their evolutionary relationships (**Figure 3A**). There are mainly two types of cis-elements in the promoter of the R-SNARE genes (**Figure 3B**): (1) plant growth and development regulatory elements, which include meristem, endosperm, circadian control regulatory elements, and palisade mesophyll cell differentiation elements; and (2) abiotic stress response elements, which include light-, cold-, drought-, auxin-, ABA-, and gibberellin-responsive elements. Detailed cis-element information is briefly summarized in **Additional File 1** (**Table S5** **)**. Approximately 71% (10) of the elements were stress response components identified by PlantCARE, which means that these R-SNARE genes might participate in cotton resistance to abiotic stress. Importantly, drought response elements were found to be present in the promoter region of many R-SNARE genes, such as *GhVAMP72e*, *GhVAMP72r*, and *GhVAMP72b*, indicating that these R-SNAREs might affect drought tolerance. Untranslated regions (UTRs) and introns usually play a role in regulating gene expression and aid in understanding gene family evolution (Rui et al., 2021). Thus, it is necessary to analyze exon/intron structures. Interestingly, except for the *GhTMS* and *GhTMSL* subgroup genes, all R-SNARE genes contained five exons. Among these genes, *GhVAMP72s* and *GhVAMP72r* have typical gene structure characteristics; however, the first exon is longer than that of other standard R-SNAREs, and this difference is likely to be formed randomly in the process of evolution (**Figure 3C**). These results indicate a strong correlation between the phylogeny and exon/intro structure, and the standard R-SNAREs were significantly conserved in cotton.

### 3.5 Analysis of protein structure

**Figure 4:**
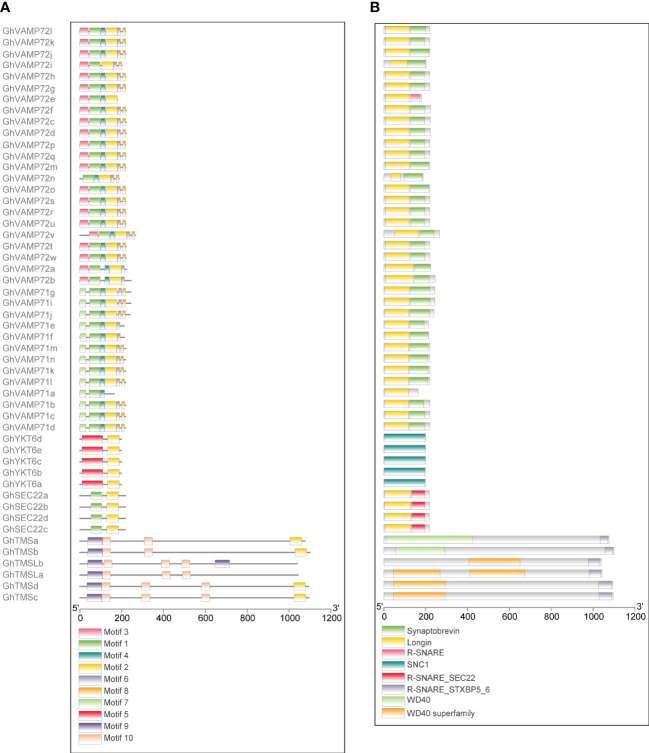
Predicted conserved motifs and protein domains of the R-SNARE family in *G. hirsutum*. The predicted conserved motifs and protein domains are presented next to their corresponding proteins. **(A)** Conserved protein motifs in the R-SNARE family identified by the MEME program. Each motif is indicated by a specific color. **(B)** R-SNARE protein domain predicted by the NCBI CDD. The protein length can be estimated by the scales at the bottom.

We used the identified protein sequences of 51 *R-SNARE* genes to further investigate the conservation of R-SNARE motifs and domains. Ten conserved motifs were detected in upland cotton R-SNARE proteins, 10 conserved motifs were detected (**Figure 4A**); information about these motifs is provided in **Additional File 2** (**Figure S1** **)**. Specifically, except for GhTMSLa/b and GhVAMP71a, the other R-SNAREs contained motif 2, which can form the R-SNARE domain. Furthermore, R-SNAREs in the same subgroups shared conserved motif compositions and protein domains (**Figure 4B**), which demonstrated that the functions of R-SNAREs in the same subfamily were similar. Notably, the N-terminal motifs and domains in the GhTMS and GhTMSL subgroups are quite different from those of other subfamilies, indicating that the functions of the GhTMS and GhTMSL subgroups are quite different from those of other subfamilies. Furthermore, we queried the SNARE domains at the C-terminus of all R-SNAREs via BLAST, and the results showed that the SNARE domains of GhTMS and GhTMSL were different from those of the other SNAREs. For all R-SNAREs, the protein identity was 44.25%, but the other SNAREs showed higher sequence similarity; the identity of other SNAREs (excluding GhTMS and GhTMSL) was 48.29% [**Additional File 2** (**Figure S2** **)**]. Therefore, based on the analysis of exon/intron structure, protein motifs and domains, we discuss only the function of standard R-SNAREs below.

### 3.6 Expression analysis of R-SNARE genes involved in drought tolerance

**Figure 5:**
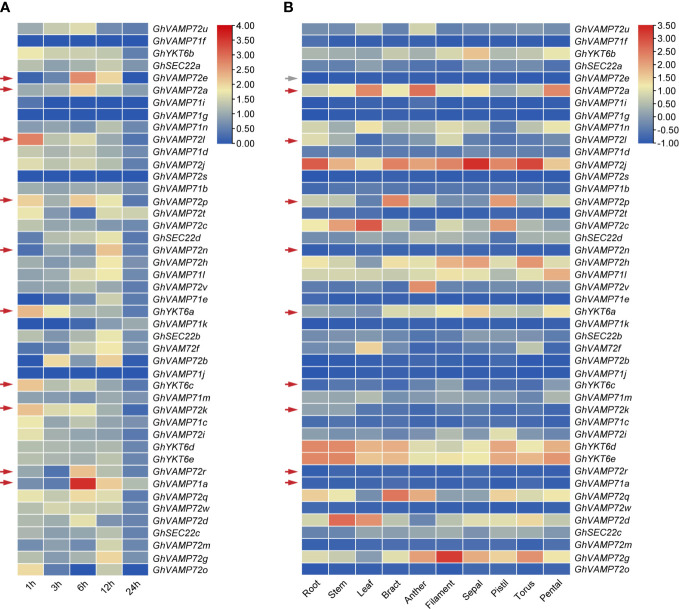
**(A, B)** Heatmap of expression patterns of *R-SNAREs* in different tissues of *G. hirsutum* under drought stress. The results of a cluster analysis of gene expression levels on different color scales are shown at the upper right. TBtools was used for the generation of heatmaps. Drought stress was imposed via PEG6000. The red arrows indicate the candidate drought-induced R-SNARE genes, and the gray arrows indicate the unexpressed genes.

To screen the drought tolerance R-SNARE genes, their transcription patterns in leaves of cotton under drought were determined by analyzing the reference genome transcriptome data of the upland cotton line ‘TM-1.’ RNA-seq analysis demonstrated that *GhVAMP72e*, *GhVAMP72a*, *GhVAMP72l*, *GhVAMP72p*, *GhVAMP72n*, *GhYKT6a*, *GhYKT6c*, *GhVAMP72k*, *GhVAMP72r*, and *GhVAMP71a* were upregulated by more than 2-fold (**Figure 5A**). Among these 10 genes, *GhVAMP72e* had a relatively low expression level in all tissues, whereas other genes were expressed at different levels in the leaves, stems, and roots (**Figure 5B**). The expression patterns of the nine selected R-SNARE genes in plants under drought stress were further investigated by RT-qPCR assays. The results showed that the expression of all nine R-SNARE genes increased to varying degrees under drought stress. *GhVAMP72l* showed maximum upregulation under drought stress, whereas *GhVAMP72n* was upregulated the least [**Additional File 2** (**Figure S3** **)**]. Based on the gene expression patterns under drought stress, we found that the nine screened R-SNARE family genes might be involved in the response to drought stress in upland cotton, and *GhVAMP72l* was selected to validate its role in drought stress tolerance.

### 3.7 Silencing *GhVAMP72l* reduces cotton resistance to drought stress

**Figure 6:**
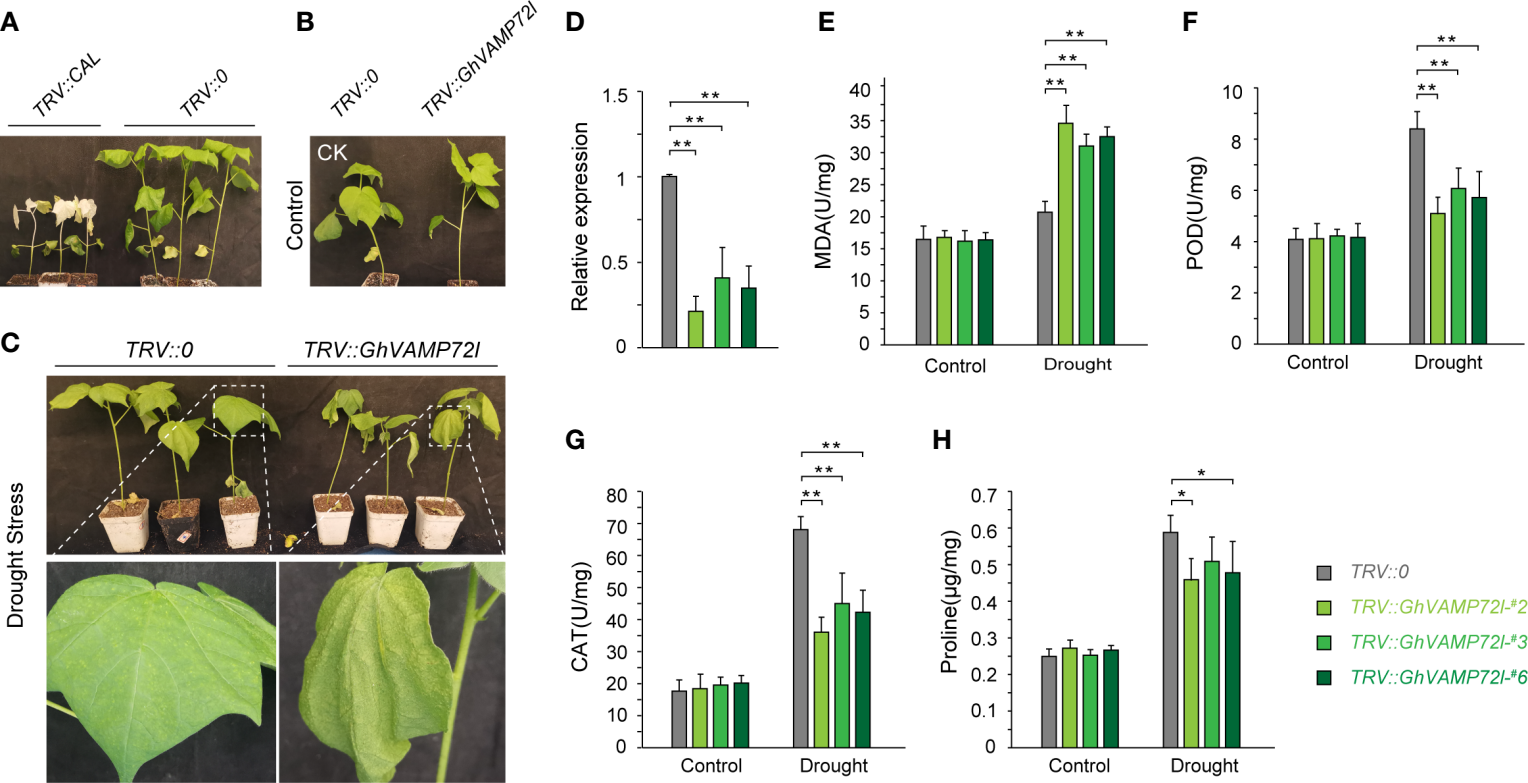
Functional validation of *GhVAMP72l* by VIGS. **(A)** Albino phenotypes of *TRV::CAL* plants. *TRV::0* served as a negative control. **(B, C)** Phenotypes of *GhVAMP72l* VIGS plants under control **(B)** and drought stress **(C)**. **(D)** Silencing efficiency of the *GhVAMP72l* gene via VIGS. **(E–H)** The MDA content, POD activity, CAT activity, and proline content in *TRV::0* and *TRV::GhVAMP72l*-silenced plants under control conditions and drought stress. * indicates a statistically significant difference between the transgenic lines and their corresponding control plants, ** indicates the highly significant difference. The values represent the means ± SDs of three independent experiments.

The function of *GhVAMP72l* under drought stress conditions in cotton was determined using VIGS technology. The ‘TM-1’ cotton lines transformed with *TRV::CLA* displayed an albino phenotype after infection, indicating that VIGS experiment was successful (**Figure 6A**). The *TRV::GhVAMP72l* and *TRV::0* plants were treated with 17% PEG6000 for 20 days, and the *TRV::GhVAMP72l* plants showed more severe wilting than did the *TRV::0* plants; *TRV::GhVAMP72l* plants and *TRV::0* plants grown under normal conditions were used as controls (**Figures 6B, C**). RT-qPCR analysis revealed that the expression of *GhVAMP72l* was significantly reduced in *TRV::GhVAMP72l* plants compared with *TRV::0* controls (**Figure 6D**). We also measured the following physiological indicators under drought treatment: MDA content, CAT activity, POD activity, and proline content. As shown in **Figures 6E–H**, unlike those in the control plants, the CAD and POD activities in the *TRV::GhVAMP72l* plants were significantly lower than those in the *TRV::0*, and the proline content was slightly lower than that in the *TRV::0* plants; however, the MDA content in the *TRV::GhVAMP72l* plants was significantly higher than that in *the TRV::0* plants. Collectively, the drought tolerance of *TRV::GhVAMP72l* plants was weaker after silencing, and *GhVAMP72l* was found to function in drought tolerance.

## 4 Discussion

### 4.1 Characterization of cotton *R-SNARE* genes

The SNARE gene family has not been extensively analyzed in plants, especially in cotton (Wang et al., 2021; Xu et al., 2022). In the present study, we used Arabidopsis R-SNARE sequences as queries to identify 51 R-SNAREs in *G. hirsutum*. The number of R-SNAREs in *G. hirsutum* was greater than those in *A. thaliana* and *O. sativa*, implying that gene duplication can result in many members (particularly in polyploid crop species) (). Phylogenetic analysis indicated that the members of the R-SNARE gene families in Arabidopsis, rice and cotton can be divided into six groups (TMS, TMSL, SEC22, YKT6, VAMP71, and VAMP72) and were named according to those in Arabidopsis (Chen et al., 2019), indicating that R-SNAREs are conserved in various species from the perspective of evolution.

Ordinary R-SNARE proteins have an N-terminal regulatory domain (called H_abc_), a coiled-coil domain (also called the R-SNARE domain) and a transmembrane domain, wherein the H_abc_ domain folds back into its helical coil structure to inhibit its activity, thereby preventing the formation of the trans-SNARE complex, and the transmembrane domain enables R-SNAREs to be anchored to the vesicle membrane (Daste et al., 2015; MartiniEre and Moreau, 2020). Specific R-SNAREs, such as TMSs and TMSLs, lack a transmembrane and H_abc_ structure but contain a WD40 regulatory domain in their N-terminus and an R-SNARE or R-SNARE-like domain in their C-terminus (MartiniEre and Moreau, 2020). Gene and protein structure analyses of upland cotton revealed that most R-SNAREs in the same group had similar gene structures and motif distributions (**Figures 3**, **4**). Nevertheless, some R-SNAREs, such as GhVAMP72e, GhVAMP72n, and GhVAM71a, have specific protein structures (**Figure 4**), whereas *GhVAMP72r* and *GhVAMP72s* contain a large first intron (**Figure 3C**). The TMS and TMSL R-SNARE domains were largely different from the ordinary R-SNARE domain; thus, we did not further investigate their functions [**Additional File 2** (**Figure S2** **)**]. The results of our analysis indicate that the structure of R-SNAREs in upland cotton is highly conserved; however, many gene duplication events have occurred during the evolution of upland cotton, which led to the generation of new R-SNARE genes.

The cis-element distribution in the promoter regions of the R-SNAREs in *G. hirsutum* was surveyed (**Figure 3B**), and a certain number of cis-elements related to drought response were observed. Studies on R-SNAREs have focused on the role of these proteins in regulating plant growth and development (Park et al., 2018). However, their role in plant resistance to drought stress has rarely been reported, and their role, especially in upland cotton, is largely unclear. Our results suggest that R-SNAREs may participate in the regulation of cotton responses to drought stress.

### 4.2 Expression patterns of R-SNARE genes

To better understand the role of *R-SNAREs* in drought stress tolerance in cotton, we investigated the expression patterns of *R-SNARE*s in leaves under drought stress using transcriptome data. Notably, 10 R-SNAREs were up-regulated under drought stress (**Figure 5A**). In addition, the expression profiles of R-SNARE genes in 10 different tissues were evaluated (**Figure 6B** **)**. Except for *GhVAMP72e*, the remaining nine drought-induced genes were expressed in the roots, leaves, and stems, all of which were drought-responsive tissues. We also measured the transcript levels of nine selected drought-induced R-SNAREs in plants under drought stress by RT-qPCR to confirm the previous transcriptome data. Except for *GhVAMP72n*, the expression of the other nine drought-induced R-SNAREs was upregulated under drought stress to varying degrees, indicating that these genes might be related to the cotton drought response, and the highest upregulated *GhVAMP72l* was selected for subsequent drought-related functional analysis.

### 4.3 Silencing of *GhVAMP72l* by VIGS reduced drought stress resistance

To investigate the roles of *R-SNAREs* in cotton drought resistance, *GhVAMP72l* was silenced using VIGS technology (Shi and Zhu, 2022). The results showed that *GhVAMP72l*-silenced plants were more sensitive to drought stress than control plants (**Figures 6B, C**), indicating that the drought resistance of *GhVAMP72l*-silenced plants was significantly reduced.

Drought stress usually disrupts the balance between intracellular ROS generation and scavenging, resulting in increased ROS and MDA concentrations (Liu et al., 2021). Therefore, scavenging ROS is crucial for plants to resist drought stress and antioxidant enzymatic systems, such as SOD and POD, are responsible for scavenging ROS. Moreover, proline accumulated in plant cells can maintain the stability of protoplast colloids and increase drought pressure (Yan et al., 2018; Sun et al., 2020). In *GhVAMP72l*-silenced plants, MDA content was found to be significantly increased under drought stress, indicating that the silenced plants suffered more severe ROS destruction. The POD and CAT activities of *GhVAMP72l*-silenced plants were lower than those of *TRV::0* plants, which indicated that the ability to scavenge toxic ROS in the silenced plants was lower than that in the control plants. The proline content in the *GhVAMP72l*-silenced plants was slightly lower than that in the *TRV::0* plants, indicating the weak ability of *GhVAMP72l*-silenced plants to regulate cell osmotic pressure. These results are consistent with previous findings showing that *GhVAMP72l* plays a role in drought resistance by regulating ROS levels. However, the detailed molecular mechanism through which *GhVAMP72l* regulates drought stress still deserves to be evaluated in further studies.

## Data availability statement

The datasets presented in this study can be found in online repositories. The names of the repository/repositories and accession number(s) can be found in the article/**Supplementary Material**.

## Author contributions

LL and BL designed research plans. Original draft was performed by BL. Data analysis and material collection were performed by GZ, YBL, and XC. HY, YL, and MZ conducted formal analysis and visualization. All authors contributed to the article and approved the submitted version.

## Supplementary material

Supplementary Figure 1 Detailed sequence of the predicted motifs

Supplementary Figure 2 Sequence alignment of C-terminal of all R-SNARE proteins in *G. hirsutum*.

Supplementary Figure 3 RT-qPCR analysis of expressions of nine candidate drought-induced R-SNAREs in root and leaf of *G.hirsutum* under drought treatments. Error bars denote the standard deviation calculated from three independent experiments.
