# Supplementary material for: Genome-wide identification of R-SNARE gene family in upland cotton and function analysis of GhVAMP72l response to drought stress
Source: Front Plant Sci. 2023 Jul 3;14:1147932. doi: 10.3389/fpls.2023.1147932 (PMC10351383; doi:10.3389/fpls.2023.1147932)
Supplement: Supplementary Figure 1 — Detailed sequence of the predicted motifs [file DataSheet_1.pdf]

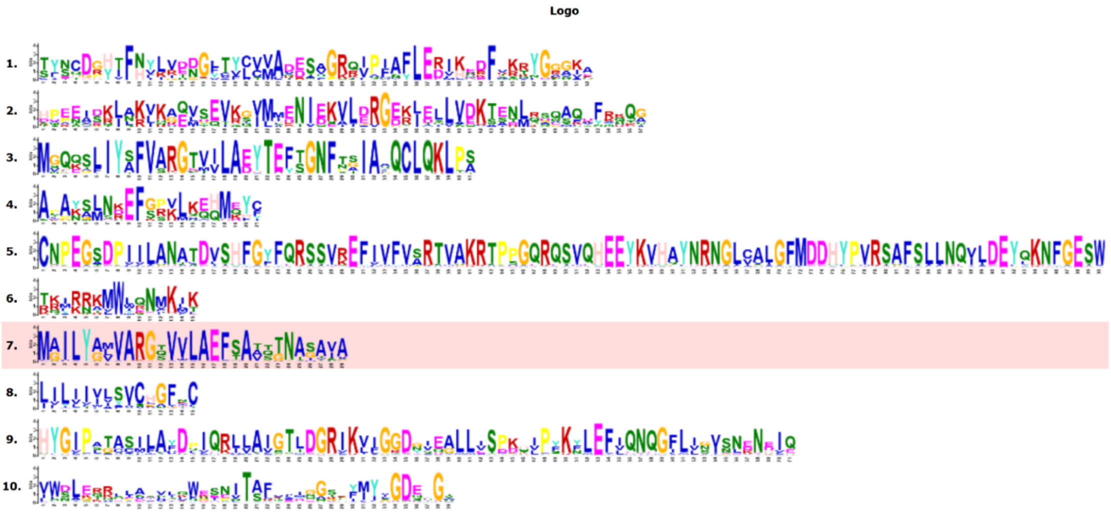

Figure S1: Detailed sequence of the predicted motifs

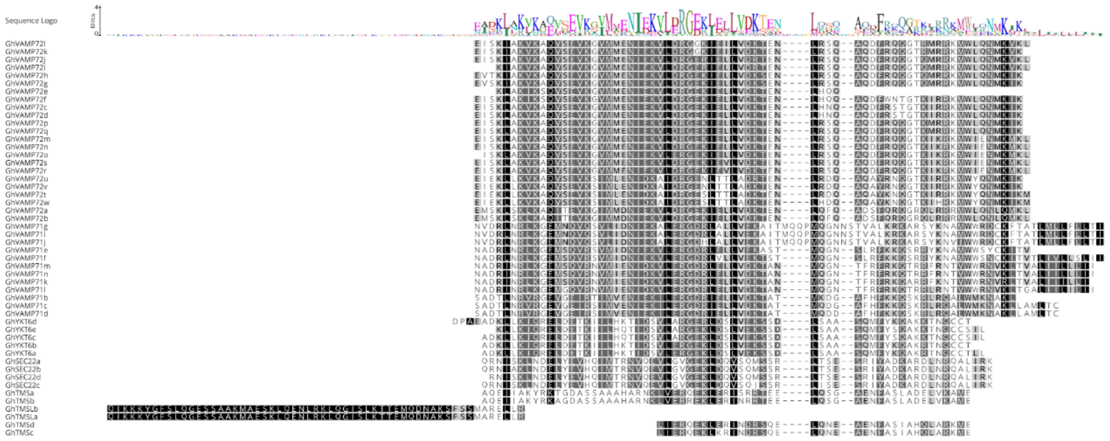

Figure S2: Sequence alignment of C-terminal of all R-SNARE proteins in *G.hirsutum*.

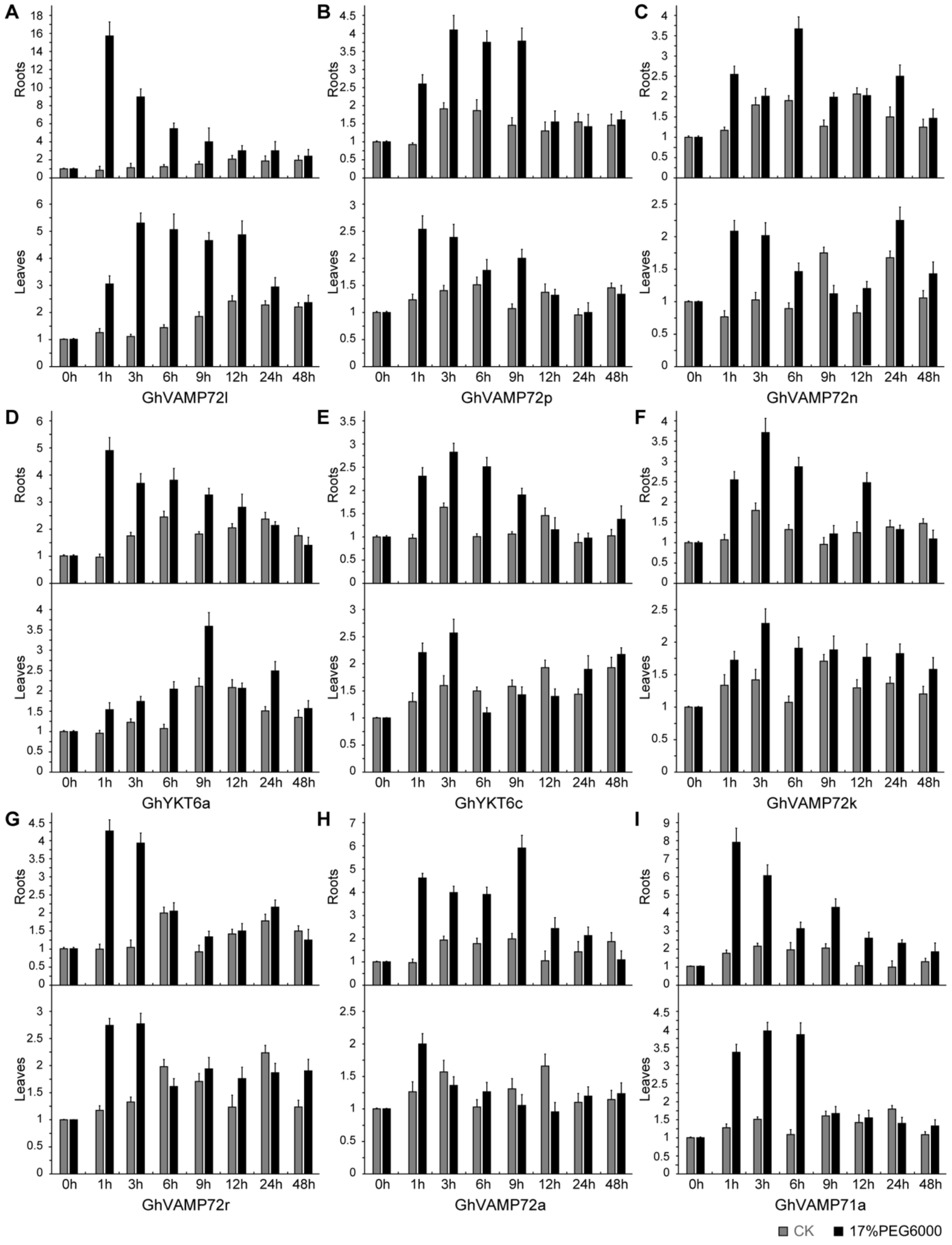

Figure S3: RT-qPCR analysis of expressions of nine candidate drought-induced R-SNAREs in root and leaf of *G.hirsutum* under drought treatments. Error bars denote the standard deviation calculated from three independent experiments.
